# Fermentation-Assisted Extraction of Isothiocyanates from Brassica Vegetable Using Box-Behnken Experimental Design

**DOI:** 10.3390/foods5040075

**Published:** 2016-11-04

**Authors:** Amit K. Jaiswal, Nissreen Abu-Ghannam

**Affiliations:** School of Food Science and Environmental Health, College of Sciences and Health, Dublin Institute of Technology, Cathal Brugha Street, Dublin D01 HV58, Ireland; amit.jaiswal@dit.ie

**Keywords:** Box-Behnken design, fermentation, isothiocyanates, lactic acid bacteria, brassica vegetables, cabbage

## Abstract

Recent studies showed that Brassica vegetables are rich in numerous health-promoting compounds such as carotenoids, polyphenols, flavonoids, and glucosinolates (GLS), as well as isothiocyanates (ITCs) and are involved in health promotion upon consumption. ITCs are breakdown products of GLS, and typically used in the food industry as a food preservative and colouring agent. They are also used in the pharmaceutical industry due to their several pharmacological properties such as antibacterial, antifungal, antiprotozoal, anti-inflammatory, and chemoprotective effects, etc. Due to their widespread application in food and pharmaceuticals, the present study was designed to extract ITCs from York cabbage. In order to optimise the fermentation-assisted extraction process for maximum yield of ITCs from York cabbage, Box-Behnken design (BBD) combined with response surface methodology (RSM) was applied. Additionally, the GLS content of York cabbage was quantified and the effect of lactic acid bacteria (LAB) on GLS was evaluated. A range of GLS such as glucoraphanin, glucoiberin, glucobrassicin, sinigrin, gluconapin, neoglucobrassicin and 4-methoxyglucobrassicin were identified and quantified in fresh York cabbage. The experimental data obtained were fitted to a second-order polynomial equation using multiple regression analysis, and also examined by appropriate statistical methods. LAB facilitated the degradation of GLS, and the consequent formation of breakdown products such as ITCs. Results showed that the solid-to-liquid (S/L) ratio, fermentation time and agitation rate had a significant effect on the yield of ITCs (2.2 times increment). The optimum fermentation conditions to achieve a higher ITCs extraction yield were: S/L ratio of 0.25 *w*/*v*, fermentation time of 36 h, and agitation rate of 200 rpm. The obtained yields of ITCs (45.62 ± 2.13 μM sulforaphane equivalent (SFE)/mL) were comparable to the optimised conditions, indicating the accuracy of the model for the fermentation-assisted extraction of ITCs. This method has good prospects in industrial applications for the extraction of ITCs, and can be helpful in the food, pharmaceutical and agricultural sectors.

## 1. Introduction

Isothiocyanates (ITCs) are a family of compounds characterised by a sulphur containing an N=C=S functional group [1]. It is an important and unique class of secondary plant products primarily derived from Brassica vegetables [2]. ITCs have a range of applications in food, pharmaceuticals and agriculture. Allyl ITCs induce cell death in both colorectal cells and prostate cancer cells [3,4] and inhibit the proliferation of various types of human cancer cells [5]. Other pharmacological properties include antibacterial activity against a range of Gram-negative and Gram-positive pathogenic bacteria [1,6], antifungal activity [7,8], goitrogenic properties [9], and they have also shown to be a potent Phase II enzyme inducer, which can protect cells against the toxic and neoplastic effects of carcinogens [10], inhibit the production of nitric oxide and inhibit the expression of inducible nitric oxide synthase that is involved in inflammation and cancer [11].

ITCs are also used as a flavouring agent in foods and an anti-fermentative agent in winemaking. Recently they were also approved as a food additive (preservative) in food applications [12], and can be used for the enhancement of the shelf-life of food products [13]. They also showed biocidal activity against insects, mollusks, aquatic invertebrates and nematodes [14] and due to these properties they are used as pesticides or herbicides in agriculture.

ITCs are breakdown products of glucosinolates (GLS) and are absent in intact vegetables [15]. Upon cellular disruption, GLS and the enzyme thioglucoside glucohydrolase, EC 3.2.3.1 (myrosinase), come in contact with each other and, in the presence of water, the enzyme immediately causes the hydrolysis of the GSL. The hydrolysis products consist of an aglycone moiety, glucose and sulphate. The aglycone moiety is unstable and rearranges to form ITCs, thiocyanates, nitriles, oxazolidinethiones and epithionitriles [16]. Plants containing GLS do not release large amounts of ITCs because myrosinase is bound to the cell walls, whereas GLS is located in the vacuoles, thereby reducing contacts [17].

In recent years, several attempts were made to extract ITCs from several sources using novel technologies. Guo et al. [18] optimised the enzymolysis conditions (pH, the addition of EDTA and ascorbic acid) for ITC production from GLS in broccoli sprouts using response surface methodology. Li et al. [19] optimised the extraction of ITCs using supercritical CO_2_ under controlled pressure and temperature from wasabi root. Parniakov et al. [20] extracted ITCs and other valuable metabolites from papaya seeds using pulsed electric energy. Li et al. [19] optimised the extraction of 4-methylthio-3-butenyl ITCs in Chinese radish roots by endogenous myrosinase hydrolysis.

Due to the proven health benefits of ITCs, and their potential applications in the food industries, the extraction of ITCs from natural sources such as cabbage could be a very cost-effective way to produce such a valuable metabolite. To obtain a maximum recovery of ITCs, it is necessary to breakdown the maximum amounts of GLS, and lactic acid bacteria (LAB) fermentation could be a valuable technological process to achieve this purpose, since it favours the hydrolysis of GLS to several potentially beneficial breakdown products [16,21,22].

As per the authors’ knowledge, there has been no reported study on the optimisation of the extraction process for ITCs from Brassica vegetables via LAB fermentation. In order to optimise the LAB fermentation–assisted extraction process for the maximum extraction yield of ITCs from Brassica vegetables, the Box-Behnken design (BBD) combined with response surface methodology (RSM) was applied. York cabbage was selected as a model food system, as it is rich in singrin, a precursor of allyl ITCs [22]. Besides, the GLS content of York cabbage was quantified and the effect of LAB fermentation on GLS was evaluated. The experimental data obtained were fitted to a second-order polynomial equation using multiple regression analysis to characterise the effect of the solute-to-liquid ratio, agitation rate and fermentation time on the yield of ITCs. The optimised ITC extraction process from this study can be used for the extraction of ITCs from Brassica vegetables with potential exploitation in a range of food and pharmaceutical industries.

## 2. Materials and Methods

### 2.1. Plant Materials and Their Preparation

Fresh Irish York cabbage (*Brassica oleracea* var. capitata alba subvar. conica) was purchased from a local supermarket in Dublin, Ireland. York cabbage utilised in the study is produced in the Dublin area, and the sample is brought from the supermarket same morning upon delivery to the superstore. Fifteen to 18 York cabbage heads (22–25 kg) were randomly selected and trimmed off their outer leaves and stem. The heads were then divided into four segments, and the central core was removed. The segments were chopped into 0.5 × 5–6 cm pieces, using a vegetable cutting machine. A pooled batch of about 15 kg cabbage was stored under dark refrigerated conditions (4 °C) as the raw material.

### 2.2. Culture

*L. plantarum* ATCC 8014 was purchased from Medical Supply Company, Dublin, Ireland. The culture was maintained at −70 °C in 20% glycerol stocks and grown in De Man, Rogosa, & Sharpe (MRS) broth (Scharlau Chemie, Barcelona, Spain) at 37 °C.

### 2.3. Inoculum Preparation

For the preparation of the inoculum, 25 mL of sterile MRS broth was inoculated with 1 mL of thawed stock culture and incubated at 37 °C for 12–14 h. This was then serially diluted 100 times to obtain working culture containing 6·log CFU/mL cells as determined by plate counts.

### 2.4. De-Sulphatation of GLS

The test samples (fermented and unfermented broth (served as control)) were centrifuged (1000 rpm, 10 min) before analysis. An aliquot (1 mL) of centrifuged sample was applied to a DEAE Sephadex A-25 column (0.5 mL) and the unbound material was removed by washing with deionised water (2 × 1 mL) and sodium acetate buffer (2 × 0.5 mL) (20 mM, pH 5.0). After washing, 75 μL purified sulphatase was added, and the columns were incubated overnight at room temperature. After overnight incubation, the desulpho-glucosinolates (dGLS) were eluted from the columns with deionised water (3 × 1 mL). The collected eluent was dried under constant N2 flow and re-dissolved in 200 mL deionised water and centrifuged prior to analysis.

### 2.5. Identification and Quantification of GLS

Analysis was performed using a Hewlett-Packard HP3 D CE capillary electrophoresis system equipped with diode array detector. All separations were performed on a fused silica capillary. The separation buffer consisted of sodium cholate (250 mM) and boric acid (200 mM) at pH 8.5. The separation was carried out at 12 kV at 60 °C. The capillary was used between each run sequentially with 1.0 M NaOH (3 min), 0.1 M NaOH (1 min), water (1 min) and separation buffer (5 min). Detection was carried out at 230 nm and 280 nm. Data processing was carried out by the use of a HP Vectra 5/100 mHz Pentium with HP Chemstation V 6.01. The quantity of the dGLS was estimated as the average between the quantities calculated from the two internal standards (glucotropaeolin and singrin), taking into account the relative response factors.

### 2.6. Estimation of Total Isothiocyanates Content

Estimation of total isothiocyanates was carried out according to the method of Zhang et al. [23]. The test samples (fermented broth and unfermented broth (control, cabbage sample submerged in blanching water)) were centrifuged (1000 rpm, 10 min) before analysis. In a 2 mL centrifuge tube, 100 μL of sample was mixed with 900 μL of 100 mM phosphate buffer (pH 8.5). Finally, 1000 μL of methanol containing 8 mM of 1,2-benzenedithiol (10 mM) were added to the centrifuge tube. The reaction mixture was incubated for 90 min at 60 °C. Afterwards, the reaction mixture was cooled at room temperature, and the absorbance was measured spectrophotometrically at 365 nm. An external standard curve was prepared from sulforaphane (in methanol) in different concentrations ranging from 2.8 to 280 μM.

### 2.7. Box-Behnken Design Experiments

RSM was applied to investigate the influence of solid-to-liquid (S/L) ratio, rate of agitation and fermentation time on the growth of *L. plantarum* using STATGRAPHICS Centurion XV (StatPoint Technologies, Inc., Warrenton, VA, USA). Box-Behnken design consists of three interlocking 22 factorial designs having points, all lying on the surface of a sphere surrounding the centre of the design. In order to statistically optimise the medium components and evaluate main effects, interaction effects and quadratic effects of the three factors on the growth of *L. plantarum*, a design with three factors and three levels including five replicates at the centre point was used. The Box-Behnken design was specifically selected since it requires fewer runs than a central composite design in cases of three or four variables. This cubic design is characterised by set of points lying at the midpoint of each edge of a multidimensional cube and centre point replicates (*n* = 5) whereas the ‘missing corners’ help the experimenter to avoid the combined factor extremes. The non-linear computer-generated quadratic model is given as:

(1) $$Y=\beta_{ο}+\overset{3}{\underset{i=0}{\sum }}\beta_{i}X_{i}+\overset{3}{\underset{j=0}{\sum }}\beta_{ii}X_{i}^{2}+\overset{3}{\underset{i=0}{\sum }}\overset{3}{\underset{j=0}{\sum }}\beta_{ij}X_{i}X_{j}$$

where *Y* is the measured response associated with each factor level combination; 0 is an intercept; *i* is the regression coefficient computed from the observed experimental values of *Y*; and *Xi* is the coded level of independent variables. The terms *Xi*, *Xj* and *Xi* represent the interaction and quadratic terms, respectively. The independent variables selected are shown in Table 1 along with their low, medium, and high levels.

### 2.8. Optimisation of LAB Fermentation for Maximum ITCs Yield

In order to perform optimisation of LAB fermentation for maximum ITCs yield, appropriate amount of York cabbage sample was mixed with water as per the nutrient illustration (Table 1) in order to achieve the required S/L ratio followed by blanching and inoculation at 5% level upon cooling. The flasks were incubated at the required agitation rate (Table 1). Three samples were harvested at the times specified by the software (Table 1) and the supernatant was analysed for ITCs content.

### 2.9. Viable Cell Counts

Viable cell counts in the fermented vegetable products (log CFU/mL) were determined by the standard plate method with MRS medium. Dilution of 1 mL broth was carried out in 9 mL MRD to plate the suitable dilution. The plates were incubated at 37 °C for 36–48 h for cell enumeration.

### 2.10. Statistical Analysis

All the experiments were carried out in triplicate and replicated twice unless stated. Results are expressed as mean values ± standard deviation (SD). Data from the BBD were subjected to a second-order multiple regression analysis using least-squares regression to obtain the estimated parameter for the mathematical model. The regression analysis and analysis of variance (ANOVA) were carried out using the STATGRAPHICS Centurion XV software.

## 3. Results

### 3.1. Identification of GLS in Irish York Cabbage

Seven different GLSs have been detected in York cabbage (Table 2). The majority of GLS present belongs to the aliphatic category; however, some of the indole GLS were also evident in York cabbage. The total GLS, which is the sum of all the individual GLS detected in York cabbage, was 14.06 ± 0.074 μg/mL, among which glucoraphanin (27.88%), glucoiberin (21.91%) and glucobrassicin (17.21%) were the dominating GLS present. Sinigrin and gluconapin were also available in significant concentrations at 13.94% and 12.66%, respectively. Kusznierewicz et al. [24] studied the GLS levels of white cabbage from different regions of Europe and found that glucobrassicin and sinigrin were the most dominating GLS in all white cabbage varieties and, depending on the origin and season, these two GLS accounted for 30%–70% of the total GLS level.

The present study showed that both glucobrassicin and sinigrin were present in significant quantities; however, their concentrations were lower than values reported in the literature [24]. Ciska and Pathak [25] found that glucoiberin and sinigrin in the aliphatic group of GLS and glucobrassicin in the indole group of GLS to be dominating the GLS content in white cabbage, and constituting up to 90% of the total GLS present. The differences in GLS levels could be due to variations in genotype, variety, season, geographic location/climate, stage of maturity and growing conditions [26,27]. The discrepancy in levels of certain GLS could be also attributed to GLS hydrolysis caused by the enzyme myrosinase during shredding of the cabbage before thermal (blanching) inactivation.

### 3.2. Effect of Fermentation on GLS 

After identification and quantification of GLS from York cabbage, fermentation was carried out to estimate its effect on GLS. To discriminate the effect of fermentation on the breakdown of GLS from that of enzymatic breakdown, the endogenous myrosinase was first inactivated by blanching treatment. Ludikhuyze et al. [28] showed that myrosinase activity from broccoli increased with increasing the temperature up to 30 °C and then decreased with a further increase in the temperature, becoming zero at 50 °C. In red/white cabbage, the optimal temperature for enzyme activity was 60 °C and it decreased with the rising temperature beyond 60 °C [29]. In order to inactivate myrosinase activity and to remove the surface microflora of York cabbage, blanching was carried out for 12 min at 95 °C before the fermentation [30].

LAB fermentation was carried out with an S/L ratio of 0.25 *w*/*v* at the agitation rate of 100 rpm for 36 h as optimised in our previous study [31]. The cabbage was analysed for unhydrolysed GLS and breakdown products in terms of ITCs content before and after fermentation. Results showed that there was some initial concentration of ITCs (16.99 μM sulforaphane equivalent (SFE)/mL) present in the unfermented sample. This was expected, as under the applied conditions of shredding, certain amounts of GLS might have been hydrolysed by native myrosinase as a result of cell damage. GLS was completely absent in the cabbage broth (initial concentration (14.06 ± 0.074 μg/mL) after LAB fermentation, which could be due to its complete breakdown to ITCs during the fermentation process. The result was further confirmed as the ITCs content increased up to 2.2 times as a result of fermentation. The results obtained in this study suggest that *L. plantarum* plays a significant role in the hydrolysis of GLS into ITCs. These findings are in line with previous studies [16] where it was shown that fermentation enhanced the content of volatile GLS breakdown products in sauerkraut, and the magnitude of such increment depended on the cabbage cultivar used, as well as on the fermentation conditions.

The results of the present work provided evidence that a high concentration of GLS breakdown products can be achieved by LAB fermentation. So the objective of this study was to develop a second-order polynomial equation to characterise the effect of the S/L ratio, agitation rate and fermentation time on ITCs yield and, for that, a three-level, three-variable Box-Behnken design was applied to statistically optimise the ITCs extraction from York cabbage.

### 3.3. Optimisation of LAB Fermentation for High ITCs Yield

Statistical design for the extraction of ITCs consisted of 17 experiments, which were carried out in random order. By employing multiple regression analysis on the experimental results, the predicted response (ITCs yield) was obtained by the following second-order polynomial equation:

8.33665 + 138.34*X_1_* + 0.3288*X_2_* − 0.054*X_3_* − 315.6*X_1_*^2^ + 2.325*X1X_2_* + 0.029*X1X_3_* − 0.01276*X_2_*^2^ + 0.000080*X_2_X_3_* + 0.000309*X_1_*^2^ (2)

A summary of the analysis of variance of the experimental results of the BBD is presented in Table 3. The F-test was used to check the statistical significance of the regression equation. The analysis of variance (ANOVA) was carried out to determine whether or not the quadratic model is significant. The *p*-values were considered as a tool to check the significance of each coefficient, which also indicated the pattern of the interactions between the variables. The smaller the *p*-values are, the bigger the significance of the corresponding coefficient, which implies that the model is suitable for use. In this case, seven effects corresponding to the S/L ratio, fermentation time, agitation rate, S/L ratio × S/L ratio, S/L ratio × fermentation time, fermentation time × fermentation time and agitation rate × agitation rate were significantly different (*p* < 0.05) (Table 3). Therefore, a small variation can alter the ITCs yield to a considerable extent.

The value of 0.0082 for lack of fit implies that it is not significant as compared to the pure error and that the model equation was adequate for predicting the ITCs yield. The fitness of the model was further confirmed by a satisfactory value of the determination coefficient, which was calculated to be 0.98, indicating that 98% of the variability in the response was predicted by the model. Furthermore, the predicted ITCs yield by the final quadratic model, along with the corresponding values observed, is given in Table 4. The agreement between the ITCs yield predicted by the model and the experimental data is good, as shown by a higher *R^2^* (98%).

The contour and three-dimensional response surfaces were generated to study the interaction among the factors studied and to visualise the combined effects of factors on the ITCs yield. The interactions between the variables can be inferred from the shapes of the contour plots. Circular contour plots indicate that the interactions between the variables are negligible. In contrast, elliptical or saddle-shaped contours indicate the evidence of the interactions [32]. The 3D response surface shows interactions of two variables, while the third factor was constant at its middle level.

**Figure 1:**
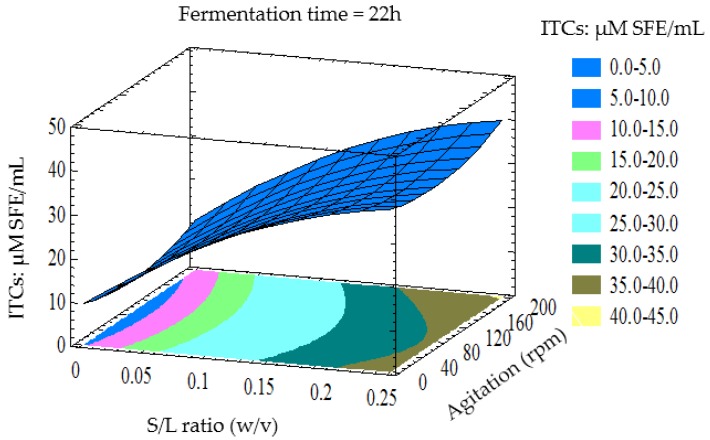
Three-dimensional response surface plot showing the effect of S/L ratio and agitation rate (rpm) on ITCs yield (ITCs: μM SFE/mL) when the response surface is fixed at fermentation time 22 h.

Figure 1 shows that the ITCs yield increased with the increment of the S/L ratio and agitation rate. The increase in the ITCs yield can be attributed to the high concentration of GLS present in the broth, and LAB activity resulted in a high yield of ITCs. The result is in line with the findings of Tolonen et al. [33]; these authors recommended the use of cultivars high in GLS to maximise the content of glucosinolate breakdown products in sauerkraut. It is hypothesised that LAB produced enzymes which were responsible for the breakdown of GLS.

**Figure 2:**
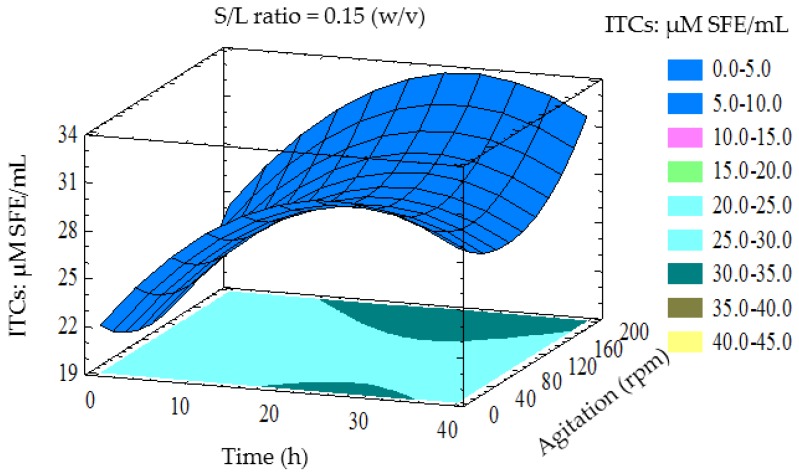
Three-dimensional response surface plot showing the effect of fermentation time (h) and agitation rate on ITCs yield (ITCs: μM SFE/mL) when the response surface is fixed at S/L ratio = 0.15.

Figure 2 shows the effect of fermentation time and agitation rate on the yield of ITCs; the S/L ratio is fixed at 0.15 *w*/*v*. The results revealed that the yield of ITCs increased as the fermentation time and agitation rates increased. The dependence of ITCs production can be understood by the fact that a high agitation rate (200 rpm) may increase the interaction of the enzyme produced by LAB with GLS, which leads to the maximum degradation of GLS and, subsequently, the production of ITCs. 

### 3.4. Verification of Predictive Model

The suitability of the model equations for predicting optimum response values was tested under the conditions: S/L ratio of 0.25 *w*/*v*, fermentation time of 36 h and rate of agitation of 200 rpm. This set of conditions was determined to be optimum by the RSM optimisation approach and was also used to experimentally validate and predict the values of the responses using the model equations. A mean value of 45.62 ± 2.13 (*n* = 3), obtained from confirmatory experiments, demonstrated the validation of the RSM model, indicating that the model was adequate for the extraction process.

## 4. Conclusions

In conclusion, RSM was successfully applied for the optimisation of the conditions for LAB fermentation–assisted extraction of ITCs from Irish York cabbage. The S/L ratio, fermentation time and agitation rate were selected as parameters influencing the ITCs yield. The high correlation of the mathematical model indicated that a quadratic polynomial model could be used to optimise the ITCs yield from plant sources. Fresh York cabbage showed the presence of several GLS such as sinigrin, gluconapin, glucoiberin, glucoraphanin, glucobrassicin, neoglucobrassicin and 4-metoxyglucobrassicin. It was observed that LAB fermentation facilitated the complete degradation of GLS and the formation of breakdown products such as ITCs. Results showed that concentration of ITCs increased more than 2.2-fold as a result of fermentation. It was concluded that *L. plantarum* may possess some enzymatic activities which hydrolyse GLS into ITCs. The present study proposes a novel biological process for the extraction of ITCs, which is highly efficient in the conversion of GLS to ITCs, and can be used by the food and pharmaceutical industries involved in the extraction of ITCs.

## Figures and Tables

**Table 1:** Level and code of independent variables used for Box-Behnken experimental design.

Independent Variables	Coded Symbols	Coded Variable Level		
		–1	0	+1
S/L ratio (*w*/*v*)	*X*_1_	0.05	0.15	0.25
Fermentation time (h)	*X*_2_	8	22	36
Agitation rate (rpm)	*X*_3_	0	100	200

**Table 2:** Glucosinolates (GLS) content in York cabbage.

Individual GLS	GLS Content (μg/mL)
Aliphatic GLS	
Sinigrin	1.96 ± 0.025
Gluconapin	1.78 ± 0.075
Glucoiberin	3.08 ± 0.070
Glucoraphanin	3.92 ± 0.178
Indol GLS	
Glucobrassicin	2.42 ± 0.047
Neoglucobrassicin	0.63 ± 0.085
4-Methoxyglucobrassicin	0.27 ± 0.038

**Table 3:** Analysis of variance of the experimental results of the Box-Behnken design (BBD).

Source	Sum of Squares	D_f_	Mean Square	F-Ratio	*p*-Value
A: S/L ratio	764.2	1	764.2	2496.9	0.0000
B: Time	24.2	1	24.2	79.0	0.0009
C: Agitation	15.6	1	15.6	50.9	0.0020
AA	41.9	1	41.9	137.0	0.0003
AB	42.4	1	42.4	138.5	0.0003
AC	0.34	1	0.34	1.12	0.3500
BB	26.3	1	26.3	86.1	0.0008
BC	0.05	1	0.05	0.17	0.7051
CC	40.2	1	40.2	131.5	0.0003
Lack-of-fit	17.1	3	5.69	18.6	0.0082
Pure error	1.22	4	0.31		
Total (corr.)	969.7	16			

**Table 4:** Box-Behnken experimental design for the three independent variables (S/L ratio, fermentation time and agitation rate), experimental and predicted values for isothiocyanates (ITCs) content.

Row	*X*_1_	*X*_2_	*X*_3_	Total ITCs Content	
	(*w*/*v*)	(h)	(rpm)	Exp	Predicted
1	0.25	36	100	36.8	38.1
2	0.25	22	200	39.7	40.4
3	0.15	8	200	28.9	29.2
4	0.15	22	100	28.7	29.0
5	0.15	8	0	24.6	26.6
6	0.05	22	200	19.2	20.3
7	0.25	22	0	38.1	37.0
8	0.15	22	100	28.3	29.0
9	0.15	22	100	29.2	29.0
10	0.05	8	100	16.4	15.1
11	0.15	22	100	29.1	29.0
12	0.15	36	0	30.1	29.8
13	0.25	8	100	29.1	28.1
14	0.05	36	100	11.1	12.1
15	0.15	36	200	34.9	32.8
16	0.05	22	0	18.8	18.1
17	0.15	22	100	29.8	29.0

(*X_1_*) S/L ratio, (*X_2_*) fermentation time, (*X_3_*) agitation rate, ITCs: μM sulforaphane equivalent (SFE)/mL.
